# Lesion Genotype Modifies High-Fat Diet Effects on Endometriosis Development in Mice

**DOI:** 10.3389/fphys.2021.702674

**Published:** 2021-09-14

**Authors:** Melissa E. Heard-Lipsmeyer, Iad Alhallak, Frank A. Simmen, Stepan B. Melnyk, Rosalia C. M. Simmen

**Affiliations:** ^1^Department of Physiology and Cell Biology, University of Arkansas for Medical Sciences, Little Rock, AR, United States; ^2^Division of Cell Biology and Physiology, Edward Via College of Osteopathic Medicine-Louisiana, Monroe, LA, United States; ^3^The Winthrop P Rockefeller Cancer Institute, University of Arkansas for Medical Sciences, Little Rock, AR, United States; ^4^Arkansas Children’s Research Institute, University of Arkansas for Medical Sciences, Little Rock, AR, United States

**Keywords:** endometriosis, high-fat diet, oxidative stress, inflammation, Krüppel-like factor 9

## Abstract

Endometriosis is a chronic, estrogen-dependent gynecologic disorder that affects reproductive-aged women and to a lesser extent, post-menopausal women on hormone therapy. The condition is associated with systemic and local immune dysfunctions. While its underlying mechanisms remain poorly understood, endometriosis has a genetic component and propensity for the disease is subject to environmental, nutritional, and lifestyle influences. Previously, we showed that high-fat diet (HFD) increased ectopic lesion numbers, concurrent with systemic and peritoneal changes in inflammatory and oxidative stress status, in immunocompetent recipient mice *ip* administered with endometrial fragments null for Krüppel-like factor 9 gene. Herein, we determined whether HFD modifies lesion parameters, when recipient peritoneal environment is challenged with ectopic wild-type (WT) endometrial fragments, the latter simulating retrograde menstruation common in women during the menstrual period. WT endometrium-recipient mice fed HFD (45% kcal from fat) showed reduced lesion incidence, numbers, and volumes, in the absence of changes in systemic ovarian steroid hormone and insulin levels, relative to those fed the control diet (CD, 17% kcal from fat). Lesions from HFD- and CD-fed recipients demonstrated comparable gene expression for steroid hormone receptors (*Esr* and *Pgr*) and cytokines (*Il-6, Il-8*, and *CxCL4*) and similar levels of DNA oxidative biomarkers. HFD moderately altered serum (3-nitrotyrosine and methionine/homocysteine) and peritoneal (reduced glutathione/oxidized glutathione) pro-oxidative status but had no effect on peritoneal inflammatory (tumor necrosis factor α and tumor necrosis factor receptor 1) mediators. Results indicate that lesion genotype modifies dietary effects on disease establishment and/or progression and if translated, could be important for provision of nutritional guidelines to women with predisposition to, or affected by endometriosis.

## Introduction

Endometriosis is a debilitating, estrogen-dependent disease that affects ~10% of reproductive-aged women (Burney and Giudice, 2012) and to a lesser extent (2–5%), post-menopausal women on hormone therapy (Secosan et al., 2020). Because the disease is latent at the very early stages, it can progress undetected until manifestation of overt symptoms which include chronic pelvic pain, dysmenorrhea, and infertility. There are currently no validated biomarkers with high sensitivity and specificity for the diagnosis of endometriosis (Burney and Giudice, 2012; Fassbender et al., 2015). Moreover, medical treatments remain ineffective, leading to its high recurrence, increased risk for ovarian and endometrial cancers, significant reduction in quality of life, and an annual economic burden of ~$50 B in the United States alone (Anglesio and Yong, 2017; Kotlyar et al., 2019; Johnatty et al., 2020; Reis et al., 2020).

Extensive research to understand the pathogenesis of endometriosis suggests the involvement of multiple predisposing genetic aberrancies, a number of which have been experimentally evaluated in mouse models and further confirmed for presence in human lesions (Hirota et al., 2008; Heard et al., 2014; Burns et al., 2018; Han et al., 2019). These genes include those involved in immune, angiogenic, steroid hormone signaling, apoptotic, and proliferative pathways (Kim et al., 2014; Brown et al., 2018). Whereas genome-wide association studies have identified several loci associated with the condition (Painter et al., 2018; Gallagher et al., 2019), the complex regulatory network underlying disease etiology precludes the identification of a single genetic “driver.” Confounding this problem is the increasingly recognized “plasticity” of endometriosis to environmental, reproductive, and lifestyle factors (Nagle et al., 2009; Matta et al., 2020). A recent study of twins in Sweden reported the heritability of endometriosis to be 47%, with the remaining effect (53%) accounted for by the environment (Saha et al., 2015). While these findings necessitate confirmation in a larger population, they reveal significant genetic and environment interactions in disease development.

The chronic inflammatory nature of endometriosis in women suggests that dietary components with pro- and anti-inflammatory actions may constitute useful targets for its prevention and treatment. Indeed, a number of recent reviews have highlighted the link between diet and endometriosis risk (Saguyod et al., 2018; Yamamoto et al., 2018). However, despite anecdotal support for (e.g., anti-oxidant rich foods; dairy products) or against (trans/saturated fats and red meat) specific food groups and nutrients in reducing endometriosis risk and progression, questions persist on the demographics of women with endometriosis who can benefit from dietary interventions. The reported inverse association between endometriosis risk and higher body fat (Shah et al., 2013), the latter generally a consequence of increased caloric intake, appears to counter the notion of obesity, a known inducer of inflammation, as predisposing to endometriosis. Moreover, potential dietary influences on subsets of women developing endometriosis are difficult to predict, given the highly variable dietary patterns of the general population and in the context of the well-accepted Sampson’s theory of retrograde menstruation, a common occurrence (> 90%) among women, as disease origin (Sampson, 1927).

In an earlier study, we showed, using an immunocompetent mouse model of endometriosis, generated with intraperitoneal administration (*ip*) of endometrial fragments null for the progesterone receptor interacting protein Krüppel-like Factor 9 (KLF9), that high-fat diet (HFD) promoted endometriosis progression in the absence of ovarian dysfunction, insulin resistance, and significant increase in bodyweight (Heard et al., 2016). Given the many genetic permutations associated with endometriosis (Méar et al., 2020), the present study addressed the question of whether HFD effects may be dependent on lesion genotype. By using HFD as a “nutritional” prototype and lesions generated from wild-type (WT) endometria as a genetic phenotype to recapitulate retrograde menstruation in normal women, we provide support for ectopic lesion genotype as an important variable in assessing dietary contributions to endometriosis risk and progression.

## Materials and Methods

### Animals

**Figure 1:**
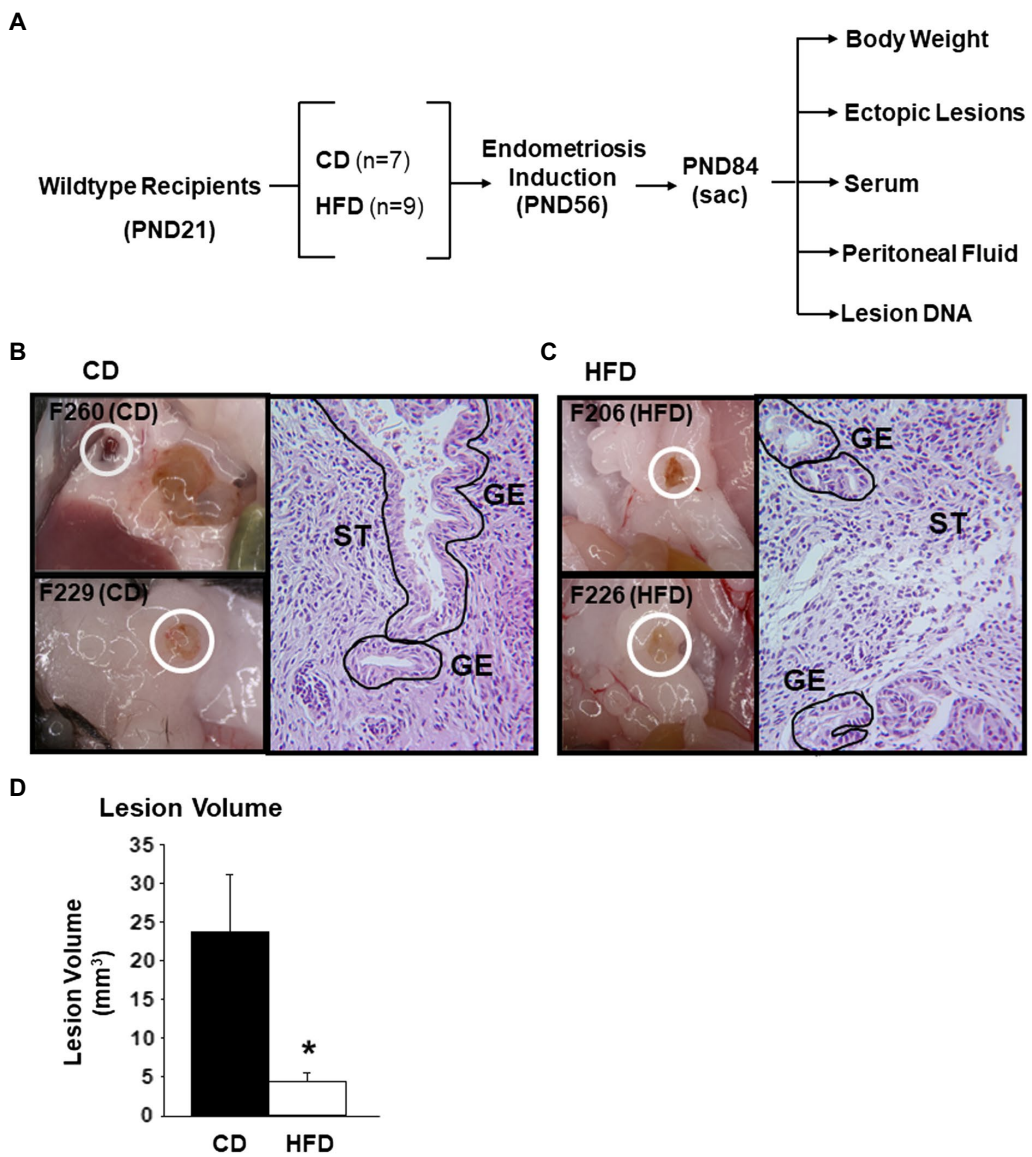
Experimental design for characterization of endometrial-like lesions derived from immunocompetent mice fed CD or HFD. **(A)** Schematic representation of generation of lesions and their analyses. Female WT mice were used as recipients of endometrial fragments from WT donors, with the indicated numbers of mice used. **(B)** Representative bright-field images of lesions from two mice (F260 and F229) fed control diet (CD) and an H&E-stained section of one lesion (F260), showing glandular epithelial (GE) and stromal (ST) cells. **(C)** Representative bright-field images of lesions from two mice (F206 and F226) fed high-fat diet (HFD) and an H&E-stained section of one lesion (F206), showing glandular epithelial (GE) and stromal (ST) cells. **(D)** Mean lesion volumes from CD-fed (*n*=7) and HFD-fed (*n*=6) recipients. ^*^p<0.05 by Student’s *t*-test.

Adult mice for breeding (C57BL/6J; Jackson Laboratory, Bar Harbor, Maine) were housed in standard cages under controlled lighting (12h light/12h dark cycle) and temperature 22C with access to food and water *ad libitum*. Pups generated from breeding pairs were weaned at postnatal day 21 (PND 21) and body weights were obtained. Females were assigned randomly to either donor or recipient groups. Donors at weaning were provided the standard American Institute of Nutrition-93G-based pelleted diet containing Casein as the sole protein source (AIN93G-CAS; Harlan-Teklad) and 17% total kcal from lard fat (Control Diet, CD). Recipients at weaning were assigned randomly to either the CD or the HFD; the latter consists of AIN93G-CAS containing 45% of total kcal from lard fat, recapitulating the percent kcal from fat in a typical “Western” diet (Wilson et al., 2007). After 5weeks on their assigned diets (at PND 56), recipients were *ip* injected with minced endometrium isolated from donors of the same age and estrous cycle stage (determined by vaginal cytology after staining with methylene blue) and continued on their assigned diets (CD or HFD). The preparation of endometrial fragments for intraperitoneal injection followed our previously described protocols (Heard et al., 2014, 2016). Briefly, the intact donor uterus was isolated, opened longitudinally by midline incision, and myometrium was scraped off the endometrium by careful upward and lateral traction. The intact endometrium was minced into ~1mm sizes by scalpel blades. All recipients received the same amount of minced tissue (40μg in 400μl of PBS) by injection into the peritoneal cavity through the abdominal wall on the ventral midline just below the umbilicus, using a syringe with a 20-gauge needle. Recipients were euthanized 4weeks later (PND 84), and body weights, sera, lesions, and peritoneal fluids were collected (Figure 1A). All procedures were in accordance with the National Institutes of Health Guidelines for the Care and Use of Laboratory Animals and approved by the University of Arkansas for Medical Sciences Institutional Animal Care and Use Committee (IACUC#3551).

### Collection, Imaging, and Morphometry of Endometriotic Lesions

Lesions in the abdominal cavity were visualized, counted, and photographed using a Carl Zeiss SteREO Discovery V8 steromicroscope (Carl Zeiss, Inc.; Oberkochen, Germany) equipped with a Canon EOS 1000D camera (Canon, Inc.). Lesion volume was calculated using a standard ellipsoid formula $D_{1}^{2}$ *×* D_2_ *×π*/6, where D represents lesion diameter and D_1_ is defined as the smaller of the two diameters (Laschke et al., 2010; Heard et al., 2014). Collected lesions were divided into two parts – one part was snap-frozen in liquid nitrogen for RNA isolation and subsequent gene expression analyses (below), whereas the other part was placed in 10% neutral-buffered formalin, paraffin-embedded, and processed for hematoxylin and eosin (H&E) staining. Images from H&E-stained sections were acquired using an Axiovert 200M microscope with an Axiocam HRc camera and Axiovision software (Carl Zeiss).

### RNA Isolation and Quantitative RT-PCR (QPCR)

Total RNA was extracted from individual lesions using the QIAGEN AllPrep kit (QIAGEN, Germantown, MD) following the manufacturer’s protocol. RNA concentrations were determined using the NanoDrop ND-1000 spectrophotometer (NanoDrop, Thermo Fisher Scientific, Waltham, MA). Reverse transcription was performed using 1μg of total RNA and the iScript cDNA Synthesis kit (Bio-Rad Laboratories, Hercules, CA). Primers (Supplementary Table 1) were designed using the Primer Express software (Applied Biosystems, Foster City, CA). An aliquot of the cDNA synthesis product was subjected to QPCR using iTaq Universal SYBR Green Supermix (Bio-Rad) with appropriate primers and the Bio-Rad CFX96 Real Time System module/c1000 Touch thermal cycler. A standard curve was generated by serially diluting pooled cDNAs (obtained in equal aliquots from all samples) beginning with the most concentrated cDNA pool designated as 10,000 arbitrary units. Target mRNA abundance was normalized to a factor derived from the geometric mean of expression values for *β-actin* and *Gapdh* mRNAs calculated using the GeNorm program (Pabona et al., 2012). The transcript levels of these reference genes did not differ between the experimental groups (Supplementary Figure 1).

### Serum RIA and ELISA

Whole blood (~500μl) was collected from non-fasted recipients by closed cardiac puncture at sacrifice. Serum was isolated from whole blood by centrifugation at 4600g for 1h and stored at −80C prior to analysis. Serum estrogen, progesterone, and insulin levels were measured using the Ultrasensitive Estradiol kit (Beckman Coulter, Brea, CA), Progesterone EIA kit (Cayman Chemicals, Ann Arbor, MI), and mouse insulin ELISA (Millipore, Inc., Burlington, MA), respectively, following the manufacturer’s protocols.

### Peritoneal Fluid Collection and ELISA

Peritoneal fluids were collected by injecting 1ml cold PBS containing protease inhibitors (Halt Protease Inhibitor Cocktail; Thermo Fisher Scientific) into the peritoneum of mice recipients before lesion isolation, using a 27-gauge needle (Thermo Fisher Scientific). Fluid was retrieved using the same syringe after gentle massaging of the peritoneum and centrifuged at 1500rpm for 10min at 4C to remove peritoneal cells. Supernatants were quantified for Tumor Necrosis Factor α (TNF) and soluble Tumor Necrosis Factor Receptor 1 (sTNFR1) levels using mouse TNFα and mouse sTNFR1 ELISAs (R&D Systems, Minneapolis, MN), respectively.

### Oxidative Stress Biomarker Analyses

Sera and peritoneal fluids of recipients (all with ectopic lesions) were evaluated for levels of oxidative (reduced and oxidized glutathione; reduced and oxidized cysteine) stress biomarkers and for measures of methylation capacity (aminothiols methionine [Met] and homocysteine [Hcy]) by HPLC with electrochemical detection (HPLC-ED), following previously published procedures (Melnyk et al., 2012). Sera were also quantified for levels of nitrosative stress biomarker 3-nitrotyrosine (Rose et al., 2012). Briefly, sera or peritoneal fluids (50μl) were treated with 10% metaphosphoric acid to precipitate protein (30min, 4C) and then centrifuged for 15min at 18000g at 4C. Resultant supernatants were filtered (0.2-μm nylon membrane), and aliquots (20μl) were analyzed by HPLC (HPLC-ED model 5200A column; 5μm, 4.6×150mm; MCM, Inc). Metabolite levels were quantified using the HPLC-ED software.

### Genomic DNA Oxidative Damage and Methylation Status

Lesion genomic DNA was evaluated for levels of 8-hydroxyguanosine, a marker of oxidative stress, following previously described protocols (Melnyk et al., 2012). Briefly, lesion DNA was isolated using the QIAmp DNA Mini kit (QIAGEN) following the manufacturer’s instructions. DNA (~1μg) was treated with ribonuclease A (Sigma) to remove any contaminating RNA and digested into component nucleotides by sequential treatments with nuclease P1, snake venom phosphodiesterase, and alkaline phosphatase. Resultant nucleotides were analyzed by LC/MS/MS. Base separation was performed with a Dionex HPLC system coupled to an electrospray ionization tandem mass spectrometer (Thermo-Finnigan LCQ) using a Phenomenex Gemini column (C18, 150×2.0mm, 3-μm particle size). DNA methylation, measured as a percentage of 5-methyl cytosine in total DNA cytosine content, was assessed using liquid chromatography combined with electrospray tandem mass spectrometry (LC/MS/MS) as previously detailed (Friso et al., 2002).

### Data Analysis

Data are expressed as mean±SEM. Statistical analyses were performed using SigmaStat software (version 3.5; Systat Software). Lesion incidence between diet groups was analyzed using the Mann-Whitney rank sum test. For all other datasets, Student’s *t*-test was used. *p*<0.05 and 0.05<*p*<0.10 were considered significant and showing tendency for significance, respectively.

## Results

### Lesion Burden in Mice With HFD Intake

To determine the effects of HFD intake in an immunocompetent mouse model of endometriosis, ectopic lesions were established from syngeneic C57BL/6J mouse endometrium. Recipients assigned to either CD (*n*=7) or HFD (*n*=9) beginning at PND 21 were *ip* injected at PND 56 with same amounts of minced endometrial fragments (40μg in 400μl PBS) isolated from 8-week old CD-fed WT mice, following previously established protocols (Heard et al., 2016). Lesion development occurred for a total of 4weeks after which time recipients were sacrificed at estrus. Figure 1A summarizes the analyses conducted on the mice and resultant lesions. Body weights were evaluated at PND21 prior to dietary assignment and at the conclusion of the study (PND84) to determine percent weight gain with diet. Lesions, sera, and peritoneal fluids were collected at study termination. Representative bright-field macroscopic images of lesions obtained from two individual recipients fed CD (Figure 1B) or HFD (Figure 1C) are shown. Lesions were found adhering to the peritoneal wall, in the abdominal fat pad, and in the intestinal mesentery, at comparable frequencies between the diet groups. Lesions between the diet groups did not differ in morphological appearances including in their gross vasculature. Hematoxylin and Eosin-stained sections of the lesions derived from mice on either diet demonstrated the presence of endometrial glands and stromal cells characteristic of human endometriotic lesions (Figures 1B,C; Burney and Giudice, 2012).

Table 1 provides a summary of lesion parameters and endocrine measurements for recipients in the two diet groups. Consumption of HFD for a total of 9weeks did not cause a significant gain in body weights (BW_PND84_−BW_PND21_/BW_PND21)_×100), when compared to those fed CD (weight gain = 60% for CD vs. 58% for HFD). Interestingly, HFD consumption decreased lesion incidence and lesion numbers (Table 1). Of the seven recipients on CD, all showed lesions (100% incidence) at the conclusion of the study; by contrast, only six of nine HFD recipients displayed lesions, with the remaining showing “floaters” (two of nine) so named for superficial, non-invasive adhesions, or absence of detectable (one of nine) lesions. The numbers of lesions per mouse also differed as a function of diet, with recipients of CD and HFD showing 2.14±0.40 and 1.17±0.17 lesions, respectively. Importantly, the mean lesion volume (representing the largest lesion for each mouse) for HFD-fed recipients was significantly lower (by ~5-fold) than their CD-fed counterparts (Figure 1D). Serum estradiol, progesterone, or insulin levels did not differ with diet among recipients (Table 1), consistent with all recipients displaying normal estrous cyclicity during the study (data not shown).

**Table 1:** Parameters of lesions and recipients exposed to CD or HFD.

	Diet		
	CD	HFD	Value of *p*
Lesion parameters			
Number (per mouse)	2.1 ± 0.4	1.2 ± 0.2^*^	0.007
Volume (mm^3^)^**^	23.8 ± 7.4	4.4 ± 1.5^*^	0.03
Incidence	100% (7/7)	75% (6/9)	0.06
Body weights (g)			
PND21	9.5 ± 1.0	11.2 ± 2.3	0.11
PND84	23.7 ± 2.3	26.4 ± 1.9^*^	0.03
Serum			
Estrogen (pg/ml)	27.0 ± 1.1	25.8 ± 4.1	0.77
Progesterone (ng/ml)	10.3 ± 1.1	10.7 ± 1.9	0.86
Insulin (ng/dl)	1.9 ± 0.3	1.8 ± 0.2	0.58
Peritoneal fluid indices			
TNFα (pg/ml)	30.1 ± 0.4	31.3 ± 0.9	0.21
sTNFR1 (pg/ml)	208.5 ± 26.7	258.4 ± 26.8	0.31

* *p≤0.05 by Student’s *t*-test*;

** *Largest lesion*.

### Gene Expression Profiles of Lesions

**Figure 2:**
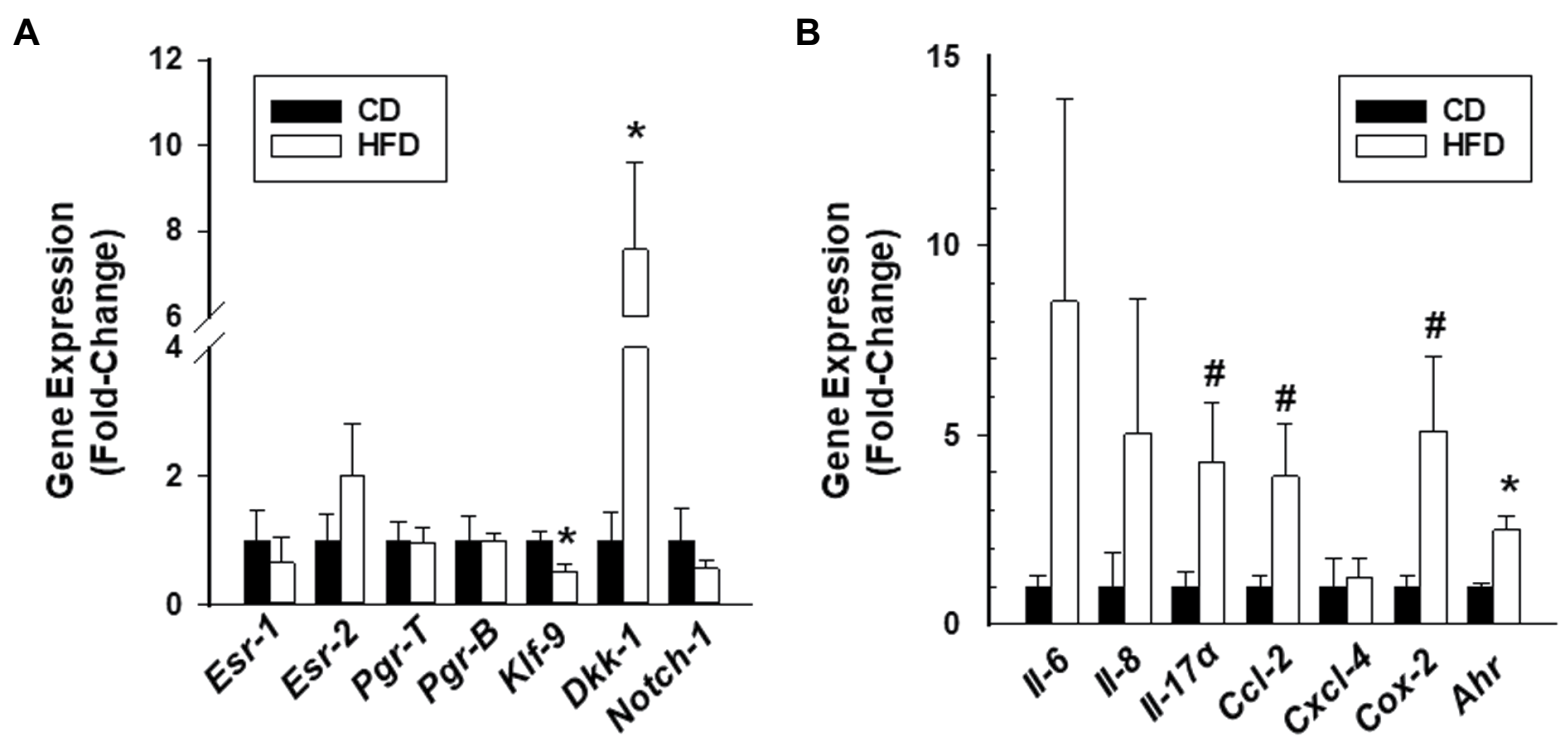
Gene expression profiles of endometrial-like lesions from recipients fed CD or HFD. Lesions were evaluated for transcript levels of steroid hormone receptor signaling components **(A)** and cytokine/inflammation-associated genes **(B)** by QPCR. Data (mean±SEM) are expressed as fold-change from CD lesion group and were obtained from *n*=7 (CD) and *n*=6 (HFD) lesions, with each lesion representing a different mouse. ^*^p<0.05 and ^#^0.10<*p*<0.05 by Student’s *t*-test.

In a previous study (Heard et al., 2016), we showed that the numbers of ectopic lesions established from endometrium lacking the progesterone receptor co-regulator KLF9 gene were increased in HFD-fed recipients, coincident with major changes in their molecular profiles. To determine whether HFD altered gene expression in lesions established from WT endometrial tissues similar to that found in *Klf9*-null lesions, we evaluated the expression of genes involved in estrogen receptor (*Esr1* and *Esr2*) and progesterone receptor (*Pgr-T, Pgr-B, Klf9, Dkk1*, and *Notch-1*) signaling pathways in lesions of recipients. Relative to CD, HFD did not induce changes in *Pgr-T, Pgr-B, Esr1, Esr2*, and *Notch-1* transcript levels but reduced and increased, respectively, *Klf9* and *Dkk1* transcripts in WT lesions (Figure 2A).

Enhanced inflammation is associated with ectopic lesion establishment and endometriotic implants synthesize and secrete a wide range of pro-inflammatory molecules (Guo et al., 2020; Heard et al., 2014; Burns et al., 2018; Vallvé-Juanico et al., 2019). Select markers were evaluated in lesions of CD-fed and HFD-fed recipients, based on their strong association with the inflammatory process (*Il-6, Il-8*, and *Cox2*; Lai et al., 2019); their previous identification as induced by HFD in *Klf9*-null lesions (*Il-17α* and *Cxcl4*; Heard et al., 2016) and the known macrophage participation in endometriosis-associated inflammation (*CxCl2*; Hogg et al., 2021). Moreover, aryl hydrocarbon receptor is an immune regulatory transcription factor (Mariuzzi et al., 2016). Relative to CD lesions, HFD lesions had higher *Ahr*, modest (but not significant) elevation in *Il17a, Ccl-2*, and *Cox2*, and comparable *Il6, IL8*, and *Cxcl4* transcript levels (Figure 2B).

### Oxidative DNA Damage in Lesions Relative to Dietary Intake

**Figure 3:**
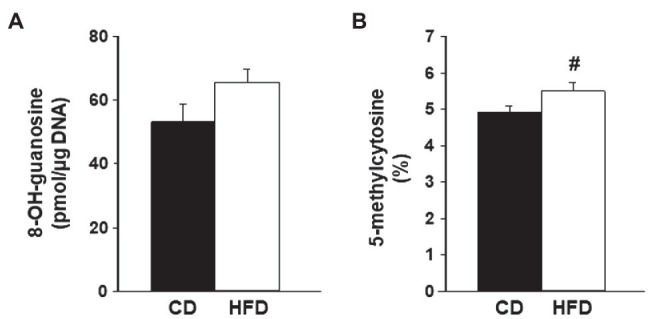
Levels of oxidative stress biomarkers in endometrial-like lesions. Genomic DNA isolated from lesions of recipients fed CD or HFD was compared for **(A)** levels of 8-OH-guanosine and **(B)** percent of methylated cytosine (5-methylcytosine) in total DNA cytosine content, following procedures described under Materials and Methods. Data (mean±SEM) are from *n*=4 individual mice per diet group. ^#^0.10<*p*<0.05 by Student’s *t*-test.

High-fat diet induces lipid accumulation in non-adipose tissues, which can lead to oxidative stress and DNA damage (Hotamisligil and Davis, 2016; Roche, 2019). To evaluate if HFD exposure promotes DNA damage in lesions generated from WT endometrium, lesion genomic DNA was assessed for changes in levels of 8-OH-guanosine, a DNA damage biomarker. The levels of 8-OH-guanosine in lesion DNA did not differ between CD-fed and HFD-fed recipients (Figure 3A).

Lesions were also compared for changes in percentages of DNA that are methylated in cytosine, a measure of DNA modification resulting from oxidative damage and considered as an epigenetic mark with regulatory potential (Scarpato et al., 2020). Lesions from HFD-fed recipients showed a tendency for increased levels of 5-methylcytosine in total DNA compared to CD lesions (Figure 3B).

### HFD Effects on Systemic and Peritoneal Redox State

**Figure 4:**
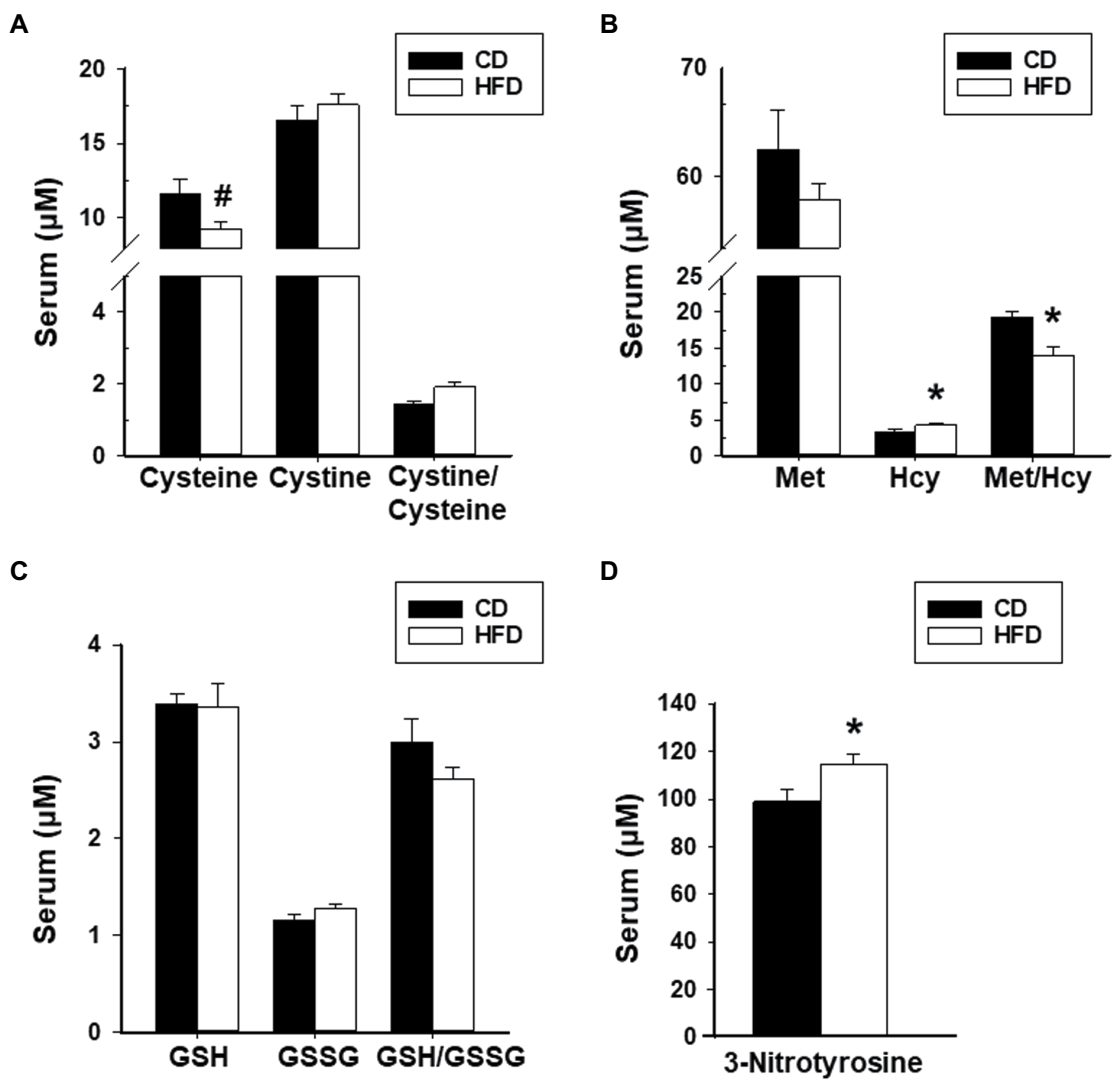
HFD effects on levels of serum oxidants and anti-oxidants and their ratios in recipient mice with lesions. **(A)** Cysteine, Cystine, and Cystine/Cysteine ratio; **(B)** Methionine, Homocysteine, and Methionine/Homocysteine ratio; **(C)** Glutathione, Glutathione disulfide, and Glutathione/Glutathione disulfide ratio; and **(D)** 3-Nitrotyrosine. Data (mean±SEM) are from *n*=7 (CD) and *n*=6 (HFD) mice per diet group. ^*^p<0.05 and ^#^0.10<*p*<0.05 by Student’s *t*-test.

**Figure 5:**
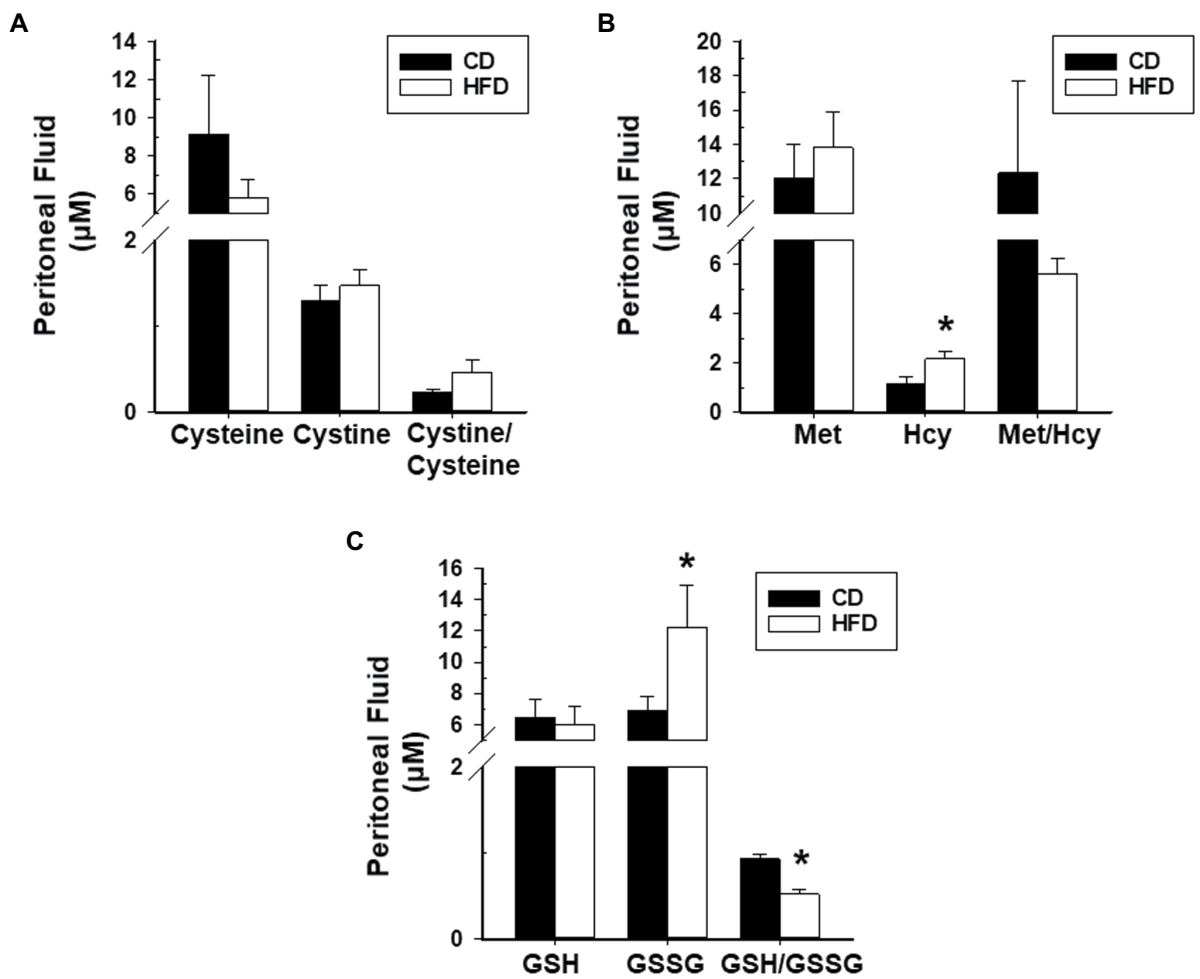
HFD effects on levels of peritoneal fluid oxidants and anti-oxidants and their ratios in recipient mice with lesions. **(A)** Cysteine, Cystine, and Cystine/Cysteine ratio; **(B)** Methionine, Homocysteine, and Methionine/Homocysteine ratio; and **(C)** Glutathione, Glutathione disulfide, and Glutathione/Glutathione disulfide ratio. Data (mean±SEM) are from *n*=7 (CD) and *n*=6 (HFD) mice per diet group. ^*^p<0.05 by Student’s *t*-test.

We next evaluated redox status in sera and peritoneal fluids of mice with WT lesions as a function of dietary intake. Sera from CD-fed or HFD-fed recipients did not differ in ratios of Cystine to Cysteine and of reduced glutathione (GSH) to oxidized glutathione (GSSG). Sera from HFD-fed recipients showed lower Met to Hcy ratio and higher 3-nitrotyrosine levels than sera from CD-fed recipients (Figures 4A–D). Peritoneal fluids from HFD-fed group showed lower GSH to GSSG ratio (due to higher GSSG levels) than the CD-fed group, and comparable Cystine to Cysteine and Met to Hcy ratios (Figures 5A–C). Measurement of peritoneal fluids for the inflammatory cytokine TNFα and its soluble inhibitor sTNFR1 also showed no differences in their levels with diet (Table 1).

## Discussion

Endometriosis is a complex and debilitating disease with no current cure, a high recurrence rate, and devastating consequences on quality of life for affected women. Recent findings indicate that both environment and genetics play critical roles in the pathogenesis of this condition (Nagle et al., 2009; Saha et al., 2015; Painter et al., 2018; Matta et al., 2020). Nevertheless, the mechanism(s) underlying potential gene/environment interactions remains poorly understood. In a previous study (Heard et al., 2016), we showed that HFD promoted the establishment of ectopic lesions from endometrial fragments lacking expression of the progesterone receptor interacting protein KLF9, in immunocompetent recipient mice. Here, we addressed whether HFD (as a dietary prototype) elicited similar effects on endometriosis progression, when lesions were initiated from WT endometria, recapitulating retrograde menstruation common to women during their periods. We found that under an identical HFD regimen, WT lesions displayed distinct behaviors from those found for *Klf9*-null lesions (Heard et al., 2016). Relative to CD-fed recipients, HFD-fed recipients with WT lesions showed a tendency for lower lesion incidence and significantly lower lesion numbers. Moreover, WT lesions of HFD-fed recipients were considerably smaller (by~5-fold) than those of CD-fed recipients, while displaying no differences in relative expression of steroid receptors *Esr* and *Pgr*. Further, the pro-inflammatory gene expression profiles of WT lesions from HFD-fed and CD-fed recipients differed only modestly (mostly non-significant), findings consistent with the limited and selective differences in the two groups’ systemic, local (peritoneal), and genomic (DNA damage) redox status. These collective results, taken together with our earlier study, highlight the significance of lesion genotype and dietary interplay and support the concept that distinct genetic dysfunctions underlying lesion establishment are an essential variable in assessing “environmental” effects on endometriosis risk and progression.

Enhanced inflammation and oxidative stress fuel each other in a vicious cycle to promote the growth and maintenance of ectopic lesions. Diet is a modifiable risk factor for many chronic diseases (Franzago et al., 2020) due to the ability of specific dietary components and metabolites to induce or suppress pro-inflammatory/pro-oxidative environments that alter cellular behaviors. A number of recent reviews have established the potential linkage between diet and endometriosis (Saguyod et al., 2018; Yamamoto et al., 2018). Based on the positive association between immune and inflammatory mechanisms and endometriosis risk, one may surmise that implementation of dietary guidelines preferential to foods that fight inflammation may be universally employed to address disease risk, progression, and recurrence. Our present data do not provide unequivocal support to this possibility since under the same HFD regimen, lesions generated from WT endometria were less invasive and displayed a reduced degree of progression (this study) when compared to lesions generated from *Klf9*-null endometria. These results suggest that genotypic differences in lesions elicit distinct responses to the inflammatory milieu generated by dietary constituents and thus, therapeutic strategies involving dietary changes in women with endometriosis may benefit from incorporating molecular analyses of retrieved lesions.

For comparison of dietary effects in an immunocompetent mouse model of endometriosis, we utilized the AIN diet containing either 17% (CD) vs. 45% (HFD) kcal from lard fat. The higher dietary fat intake (9weeks duration) of HFD-fed recipients did not result in differences in percent body weight gain, endocrine parameters (insulin, estradiol, and progesterone), and estrous cyclicity, relative to CD-fed recipients, indicating the preferential contribution of the “local” peritoneal environment rather than the systemic milieu to underlie the differences in lesion parameters. Expression of systemic inflammatory mediators is normally elevated in response to HFD (Roche, 2019). Relative to WT lesions of CD-fed recipients, those of HFD-fed recipients displayed modest increases in inflammatory status as manifested by tendencies for higher expression of several cytokines and inflammatory components (*IL17α, Ccl-2, Cox-2*, and *Ahr*). Oxidative stress status was also elevated to some extent as shown by increased levels of systemic (serum 3-nitrotyrosine), genomic (percent lesion DNA 5-methycytosine levels), and peritoneal (lower GSH/GSSG ratio) redox parameters. Nevertheless, these changes were highly attenuated relative to those observed in *Klf9*-null lesions under the same HFD regimen (Heard et al., 2016). We posit that recently described functions of KLF9 in mediating inflammatory responses underlie the differences. Studies indicate KLF9 as a key feedforward regulator of response to glucocorticoid activity (Gans et al., 2020), consistent with it being a glucocorticoid-induced gene (Bonett et al., 2009). In the context of a heightened inflammatory milieu generated by HFD, KLF9 normally expressed in endometrial fragments may promote the production of pro-inflammatory genes that hinder tissue implantation into the peritoneum. Additionally, endometrial KLF9 may support the recruitment within implantation sites, of immune cells that can enable the rapid clearance of lesions, thus, preventing progression to bigger lesions. The latter possibility is consistent with the report that KLF9 acts as a transcriptional mediator of glucocorticoid-regulated, KLF9 family member KLF2 in macrophages to promote inflammation (Chinenov et al., 2014).

Macrophages are immune cells that are highly linked to the pathophysiology of endometriosis and tumor progression (Bacci et al., 2009; Zhu et al., 2017). In a recent elegant study, Hogg et al. (2021) reported that macrophages exhibit a dual role in endometriosis, as an inhibitor and an enhancer, depending on their origins. By dissecting the macrophages that populate peritoneal ectopic lesions in a mouse model of induced endometriosis, the authors showed that tissue-resident macrophages, such as those derived from endometrial lining (“endometrial macrophages”), promote the growth of lesions while monocyte-derived macrophages in the peritoneal cavity are protective against lesion development (Hogg et al., 2021). Thus, we speculate that the basal levels of tissue-resident macrophages in WT and *Klf9*-null endometria may contribute to the distinct lesion parameters associated with the same HFD environment. Additional studies are warranted to address how loss of endometrial KLF9 may alter the ontogeny and function of endometrial-resident macrophages and the consequences of ectopic lesions formed from *Klf9*-null endometrium on the recruitment of monocyte-derived macrophages.

Epidemiological data have demonstrated an inverse association between adult BMI and the incidence of endometriosis (Shah et al., 2013). However, emerging studies have provided caveats to this relationship. One study showed that the linkage of adiposity and endometriosis is complex and maybe dependent on disease severity (Byun et al., 2020). In a prospective study of a large number of pre-menopausal women followed for a duration of 20years, greater consumption of red meat, which was associated with higher caloric intake and hence, higher BMI, was identified as a risk factor for endometriosis (Yamamoto et al., 2018). Further, findings in a mouse model have raised the possibility of endometriosis as causal to rather than a consequence of loss of body weight and body fat, due to accompanying disruptions of hepatic metabolic gene expression attributed to changes in adipocyte miRNA and stem cell numbers (Goetz et al., 2016; Zolbin et al., 2019). While CD-fed and HFD-fed recipients in our study did not differ in percent weight gains over the course of the experiment, most likely because HFD intake was limited in duration, obesogenic status (high BMI) and a HFD regimen share common inflammatory pathways. Thus, the attenuated lesion incidence and largely comparable expression of molecular parameters that are normally positively associated with endometriosis, in HFD-fed relative to CD-fed recipients demonstrated here, suggest congruence to the inverse relationship between endometriosis risk and high BMI. If so, one can infer that the inverse association is only applicable in a subset of women who may normally experience retrograde menstruation but have no genetic predisposition to endometriosis. For women with inherent (known or unknown) genetic aberrancies predisposing to the condition, the association may not be relevant due to additional influences conferred by the environment and lifestyle-induced metabolic changes (i.e., gene/environment interactions).

The present study, taken together with the earlier study using lesions of a different genotype (Heard et al., 2016), provides significant insight on the potential effects of genotype vs. environment (diet) in the establishment and progression of endometriosis, which is highly challenging to appraise in women. Nevertheless, it is important to note that our mouse model of endometriosis does not fully recapitulate the early steps in lesion establishment in women. In the latter, endometriotic lesions are widely considered to be generated from retrograde menstrual fragments which implant in the pelvic cavity or extra-pelvic sites. Our mouse model used minced endometrial tissues from syngeneic donor mice that were *ip* injected in recipient mice. Moreover, endometriotic lesions in women are classified into three histopathologic and molecular entities, namely, peritoneal, ovarian, and deep-infiltrating (Nisolle and Donnez, 1997; Zhou et al., 2020). Our mouse ectopic lesions are more akin to peritoneal lesions and are, thus, limited in relevance to the other types. Since a majority of patients at a young age who show symptoms of endometriosis and exhibit the peritoneal subtype, data presented here may still be important to the early phase of ectopic lesion establishment, rather than to the proliferative, more progressive state of the condition. Differences in the proportion of stromal vs. epithelial cells present in isolated lesions may account for the large variations in mean values for a number of reported outcomes. However, we did not find significant histological differences (predominance of stromal vs. epithelial or *vice-versa* or gross changes in vasculature) in our evaluated lesions. The lack of a reliable mouse model that faithfully mimics the human disease scenario whose pathophysiology is driven by genetic, hormonal, lifestyle, diet, and immune components is a limitation that remains a challenge to the endometriosis field.

In summary, we found that increased dietary fat intake reduced lesion incidence and lesion parameters in an immunocompetent mouse model, when ectopic implants are generated from endometrial cells lacking genetic aberrancies. When taken together with our earlier study using *Klf9*-null mutant lesions under the same dietary regimen (Heard et al., 2016), our findings suggest that dietary influences on endometriosis progression may be dependent in part, on lesion genotype. Our studies raise the intriguing possibility that integrating transcriptional profiling of excised lesions with nutritional strategies may provide a personalized approach to mitigate endometriosis progression and recurrence. We posit that further analyses of additional mouse models of endometriosis with specific lesion genotypic aberrancies, in the context of specific diets, may improve current understanding of genetic and environmental interplay in this condition. Lastly, genotypic analyses of refluxed menstrual tissues from women with endometriosis concordant with analyses of their dietary patterns (or in the context of other environmental insults) may offer a physiological correlate between the immune system and diet-induced metabolic changes associated with disease pathogenesis.

## Data Availability Statement

The original contributions presented in the study are included in the article/Supplementary Material, and further inquiries can be directed to the corresponding author.

## Ethics Statement

The animal study was reviewed and approved by the University of Arkansas for Medical Sciences Institutional Animal Care and Use Committee.

## Author Contributions

MH-L, FS, and RS conceived and designed the study and wrote, reviewed, and edited the manuscript. MH-L, IA, and SM performed the experiments and analyzed the data. RS supervised all aspects of the project and acquired funding for the study. All authors read and approved the final manuscript.

## Funding

Funding for the study was provided in part by the National Institutes of Health (grant no. RO1 HD21961), University of Arkansas for Medical Sciences (UAMS) Development Enhancement Award for Proposals (DEAP), and the UAMS Sturgis Diabetes Foundation.

## Conflict of Interest

The authors declare that the research was conducted in the absence of any commercial or financial relationships that could be construed as a potential conflict of interest.

## Publisher’s Note

All claims expressed in this article are solely those of the authors and do not necessarily represent those of their affiliated organizations, or those of the publisher, the editors and the reviewers. Any product that may be evaluated in this article, or claim that may be made by its manufacturer, is not guaranteed or endorsed by the publisher.

## Supplementary Material

Supplementary Figure 1 Expression levels of β-actin (Actb) and Gapdh mRNAs in endometrial-like lesions from recipients fed CD or HFD. Data (mean±SEM) are expressed as fold-change from CD lesion group and were obtained from *n*=7 (CD) and *n*=6 (HFD) lesions, with each lesion representing a different mouse.
