# Supplementary material for: Lesion Genotype Modifies High-Fat Diet Effects on Endometriosis Development in Mice
Source: Front Physiol. 2021 Sep 14;12:702674. doi: 10.3389/fphys.2021.702674 (PMC8547326; doi:10.3389/fphys.2021.702674)
Supplement: Supplementary file 1 [file Table_1.docx]

| *Mouse gene* | **Forward primer (5’-3’)** | *Reverse primer (3’-5’)* |
| --- | --- | --- |
| *Gapdh* | CTTTGTCAAGCTCATTTCCTGG | TCTTGCTCAGTGTCCTTGC |
| *Actb* | ACCTTCTACAATGAGCTGCG | CTTTAGCACGCACTGTAGTTT |
| *Esr-1* | GTCCTGCGAAGGCTGCAA | CCTCCGGTTCTTGTCAATGGT |
| *Esr-2* | GCCAGCCCTGTTACTAGTCCAA | TCACAGGACCAGACACCGTAAT |
| *Pgr-T* | GCTTGCACGCTTGGACTCA | GAAAAAGCAGCCCGTCCAG |
| *Pgr-B* | GGAGATAGCGGGAGTCCTTTTT | GGTCTGAGAACTGGGAGTTATTCG |
| *Klf-9* | GGCTGTGGGAAAGTCTATGG | AGTGTGGGTCCGGTAGTG |
| *Dkk-1* | ATATCCCAGAAGAACCACACTG | ATCTTGGACCAGAAGTGTCTTG |
| *Notch-1* | GCAACTGTCCTCTGCCATATAC | GTCTTCAGACTCCTTGCATACC |
| *Il-6* | CAAAGCCAGAGTCCTTCAGAG | GTCCTTAGCCACTCCTTCTG |
| *Il-8* | TGCTAGTAGAAGGGTGTTGTGCGA | TCCCACACATGTCCTCACCCTAAT |
| *IL17α* | CGCAATGAAGACCCTGATAGAT | CTCTTGCTGGATGAGAACAGAA |
| *Ccl-2* | GTCCCTGTCATGCTTCTGG | GCTCTCCAGCCTACTCATTG |
| *Cxcl-4* | CTCATAGCCACCCTGAAGAATG | AGGCAGCTGATACCTAACTCT |
| *Cox-2* | CCCCTCCTGCGAAGTTTAATC | GCTTCCCAGCTTTTGTAACCAT |
| *Ahr* | CCCCGAATCCCAACCTTTCTATG | CTTCAGCAGCAGCGTTATCT |

**Supplementary Table 1: Primer Sets**
