# Supplementary figures and images for: Lesion Genotype Modifies High-Fat Diet Effects on Endometriosis Development in Mice
Source: Front Physiol. 2021 Sep 14;12:702674. doi: 10.3389/fphys.2021.702674 (PMC8547326; doi:10.3389/fphys.2021.702674)

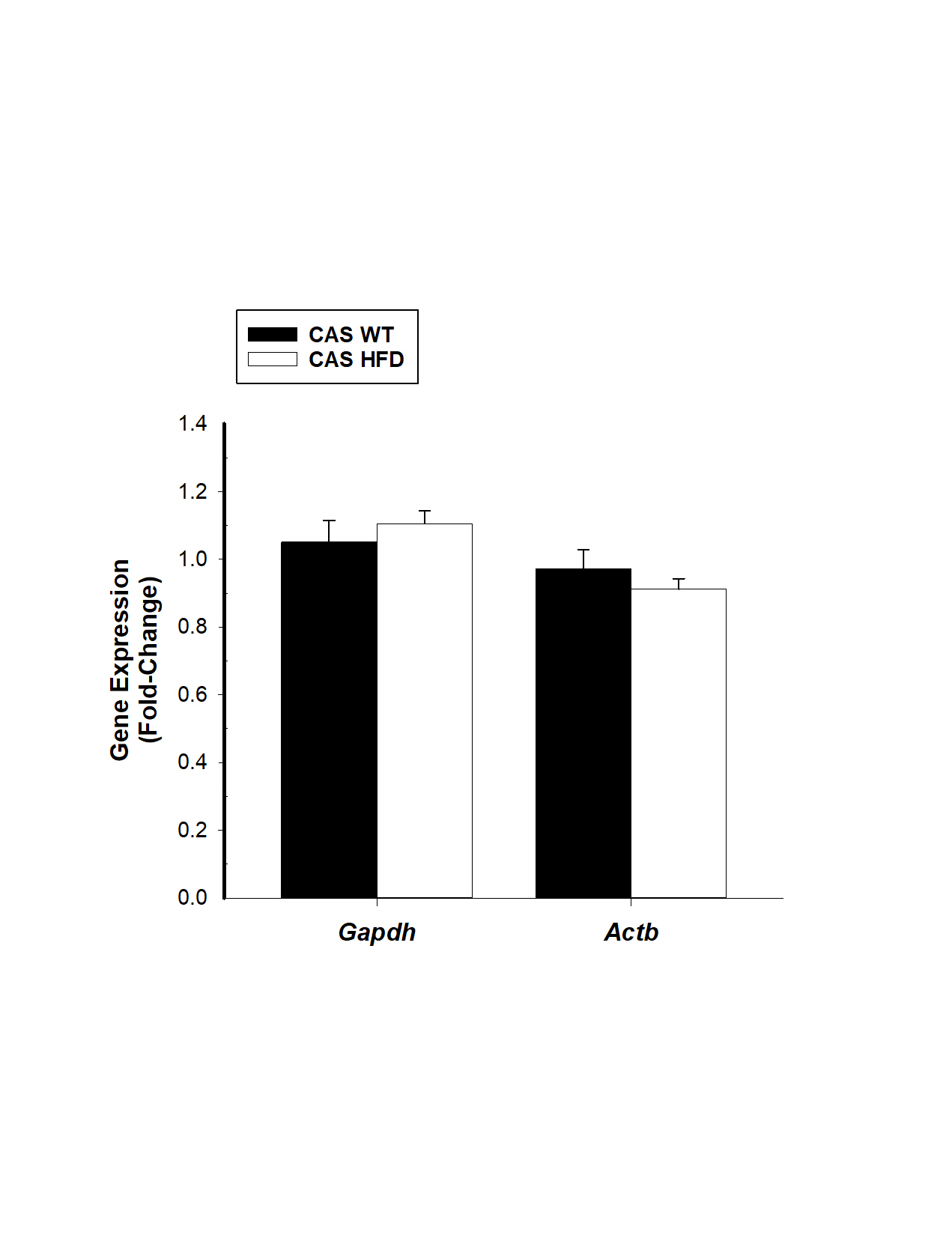

Supplement: Supplementary Figure 1 — Expression levels of β-actin (Actb) and Gapdh mRNAs in endometrial-like lesions from recipients fed CD or HFD. Data (mean±SEM) are expressed as fold-change from CD lesion group and were obtained from n=7 (CD) and n=6 (HFD) lesions, with each lesion representing a different mouse. [file Image_1.JPEG]
